# Review of the Medical Student Performance Evaluation: analysis of the end-users’ perspective across the specialties

**DOI:** 10.1080/10872981.2021.1876315

**Published:** 2021-02-19

**Authors:** Jeffrey B. Bird, Karen A. Friedman, Thurayya Arayssi, Doreen M. Olvet, Rosemarie L. Conigliaro, Judith M. Brenner

**Affiliations:** a Barbara Zucker School of Medicine, Hofstra/Northwell, Hempstead, New York, USA; b Vice Chair for Education, Department of Medicine, Northwell Health, Manhasset, NY, A Professor of Medicine at the Donald and Barbara Zucker School of Medicine, Hofstra/Northwell, Hempstead, New York, USA; c Senior Associate Dean, Medical Education and CPD, Weill Cornell Medicine-Qatar and a Professor of Clinical Medicine, Weill Cornell Medicine, NY, NY; d Assistant Professor and Medical Education ProjectManager Department of Science Education, Donald, Barbara Zucker School of Medicine, Hofstra/Northwell, Hempstead, New York, USA; e Professor of Clinical Medicine, New York Medical College; f Associate Dean for Educational Data and Analytics Donald and Barbara Zucker School of Medicine at Hofstra/Northwell, Hofstra University, Hempstead, NY, USA

**Keywords:** MSPE, residency application, educational handoff, dean’s letter, assessment data, undergraduate medical education, ume

## Abstract

The Medical Student Performance Evaluation (MSPE) is an important tool of communication used by program directors to make decisions in the residency application process. To understand the perspective and usage of the MSPE across multiple medical specialties now and in anticipation of the planned changes in USMLE Step 1 score-reporting. A survey instrument including quantitative and qualitative measures was developed and piloted. The final survey was distributed to residency programs across 28 specialties in 2020 via the main contact on the ACGME listserv. Of the 28 specialties surveyed, at least one response was received from 26 (93%). Eight percent of all programs (364/4675) responded to the survey, with most respondents being program directors. Usage of the MSPE varied among specialties. Approximately 1/3 of end-users stated that the MSPE is very or extremely influential in their initial screening process. Slightly less than half agreed or strongly agreed that they trust the information to be an accurate representation of applicants, though slightly more than half agree that the MSPE will become more influential once USMLE Step 1 becomes pass/fail. Professionalism was rated as the most important component and noteworthy characteristics among the least important in the decision-making process. Performance in the internal medicine clerkship was rated as the most influential while neurology and psychiatry performances were rated as less influential. Overwhelmingly, respondents suggested that including comparative performance and/or class rank would make the MSPE more useful once USMLE Step 1 becomes pass/fail. MSPE end-users across a variety of specialties utilize this complex document in different ways and value it differentially in their decision-making processes. Despite this, continued mistrust of the MSPE persists. A better understanding of end-users’ perceptions of the MSPE offers the UME community an opportunity to transform the MSPE into a highly valued, trusted document of communication.

## Introduction

The residency selection process has never been so complex. Residency program leadership must sift through myriad resources about potential applicants in their decision-making process regarding whom to invite and rank for residency slots. In recent years, the total number of applicants, as well as the average number of applications per medical school graduate have increased in all specialties, making the process increasingly onerous [1]. This increase is occurring when programs are being asked to review applicants in a more holistic manner, and this year, to interview candidates virtually [2–6]. Concordant with the suggestion of holistic review is the recent announcement from the US Licensing Medical Exam (USMLE) that Step 1 scores will soon be reported as pass/fail [7]. Despite the fact that USMLE Step 1 was designed as a licensing exam, it is common practice for program directors to use the score as a means of comparing candidates to one another [8]. Thus, the decision to change to pass/fail score reporting effectively removes one of the objective measures residency directors use to assess medical students.

The primary method by which undergraduate medical education (UME) institutions communicate with the graduate medical education (GME) community about student applicants is via the medical student performance evaluation (MSPE). The MSPE is one of the several resources used by program directors and others to make decisions regarding both interviewing and ranking candidates [9]. The MSPE typically contains six sections: identifying information, noteworthy characteristics, academic history, academic progress, summary, and medical school information. In 2016, with the goal of making the MSPE a better communication tool, the Association of American Medical Colleges (AAMC) MSPE Task Force recommended changing its format [10]. It addressed issues of the MSPE’s purpose, length, format and content, with a focus on increased transparency and standardization [10]. Since the initial recommendations, the majority of medical schools have adopted them [11] although there is still significant variability in the format of the MSPE across medical schools [12].

As a tool of communication between UME and GME, an important consideration for the MSPE is the perspective of the end-user, defined as a person who uses the document in the residency selection process. In 2019, we reported on a survey of MSPE end-users in Internal Medicine (IM) [13]. Those findings indicated that the MSPE provided valuable information to end-users in their applicant selection process. We have extended these findings by surveying end-users across 28 specialties to better understand how end-users from different specialties use the data included in the MSPE. To our knowledge, this is the first investigation of end-users’ perceptions of the MSPE across specialties. We hypothesized that different specialties utilize the MSPE for different purposes and at different points in the application process. We also investigated the perceived impact the proposed change in USMLE Step 1 score-reporting might have on the influence of the MSPE.

## Methods

### Survey construction

The authors used responses from a survey distributed to IM programs directors to inform the development of a pilot survey. In addition to items on the utility of each section of the MSPE, questions about professionalism and the graphic presentation of clerkship grades were included. Items in the pilot survey consisted of both closed- and open-ended questions. A pilot survey was distributed via email to the GME community from the authors’ home institutions (Northwell Health, New York Presbyterian Hospital, Westchester Medical Center). The email included a description and an anonymous link to the survey, which was administered through Qualtrics^TM^.

The authors reviewed the results from the pilot survey (60 responses) and agreed on the final version of the survey through an iterative process (Appendix 1). Three sections of questions resulted: *Influence and Usage, Areas of Importance*, and *Suggestions for the future*. During the time between the administration of the pilot survey and development of the final version, the USMLE announced the planned change in score-reporting for the Step 1 exam [7]. Therefore, questions directed at understanding how this change would alter the weight of the MSPE in the decision-making process were added, including an open-ended question addressing what additional information should be included in the MSPE in order to make it more useful to end-users. Closed-ended questions used a 5-point Likert scale asking participants to rate how strongly they agreed with statements, how much each section of the MSPE influences their decisions, or if they wait for the MSPE to be released prior to screening or inviting candidates. The professionalism questions were changed from open- to closed-ended, using the agreement 5-point Likert scale. Otherwise, closed-ended questions were categorical or multiple choice.

### Survey distribution

To prepare for distribution of the survey to a wide audience, a panel of recipients was created using the 2019–20 program lists by specialty reports from the Accreditation Council for Graduate Medical Education (ACGME) website [14]. All GME programs in 28 specialties with an email on file were compiled to create a total panel of 4675 US programs. The survey was distributed using Qualtrics^TM^ in early 2020. A reminder was sent 10 days after the initial distribution, and 10 days following that. A single response per institution was included in the final analysis.

### Data interpretation

Descriptive statistics are presented as the percent of respondents who chose the top two highest anchors on 5-point Likert questions. The data is presented as all respondents, as well as specialty-specific for the core clerkships (family medicine (FM), internal medicine (IM), neurology, obstetrics/gynecology (OB/GYN), pediatrics, psychiatry, and surgery) and specialties with a response rate of at least 15% of the programs surveyed. Two of the authors (JB and JBB) independently used content analysis to determine the presence of themes. Any differences were reconciled via conversation between JB and JBB. The data presented come from the final survey; pilot survey data is not included. Results of content analysis are presented by frequency of response.

## Results

Of the 28 specialties surveyed, at least one response was received from 26 (93%) of them. A total of 364/4675 programs (8%) responded to the survey. Of the respondents, 24 (7%) were not directly involved in reviewing MSPEs and were thus removed from the analysis, leaving 340 included in the overall analysis. Of all programs listed on the ACGME website, response rates were the highest for physical medicine and rehabilitation (PM&R) (19%), emergency medicine (EM) (18%), pediatrics (15%), anesthesiology (13%), and OB/GYN (11%) (Table 1). The majority of end-users who responded to the survey were program directors (89%); other respondents were program administrators (6%), associate program directors (2%), core faculty (2%), and department chairs (1%).

**Table 1.** Number and percent response rate by program specialty of all U.S. programs surveyed

Specialty	Number of responses by specialty	Number of programs in panel	Percent response rate by specialty
Allergy And Immunology	1	66	2%
Anesthesiology	18	143	13%
Cardiovascular Disease	2	214	1%
Critical Care Medicine	1	36	3%
Dermatology	6	125	5%
Emergency Medicine	41	233	18%
Endocrinology, Diabetes, And Metabolism	3	123	2%
Family Medicine	30	627	5%
Gastroenterology	2	162	1%
Internal Medicine	39	497	8%
Neurological Surgery	4	100	4%
Neurology	11	148	7%
OBGYN	29	267	11%
Ophthalmology	9	110	8%
Orthopedic Surgery	12	180	7%
Otolaryngology	5	111	5%
Pathology-Anatomic and Clinical	12	131	9%
Pediatrics	28	193	15%
Physical Medicine and Rehabilitation	15	78	19%
Psychiatry	18	232	8%
Radiation Oncology	6	80	8%
Radiology-Diagnostic	15	177	8%
Rheumatology	4	92	4%
Sports Medicine	4	126	3%
Surgery	19	296	6%
Urology	6	128	5%
**Total**	**340**	**4675**	**7%**

### Influence and usage

Approximately one third of all end-users stated that the MSPE is very or extremely influential in their initial screening process (32%), granting invitations for interviews (39%), and rank list decisions (35%). However, the priority of the MSPE in these roles varied amongst the specialties (Table 2).

**Table 2.** Overall and specialty specific attitudes towards the usage and influence of the MSPE. Data presented represents the percent of respondents who chose the top two highest anchors on the respective 5-point Likert scale

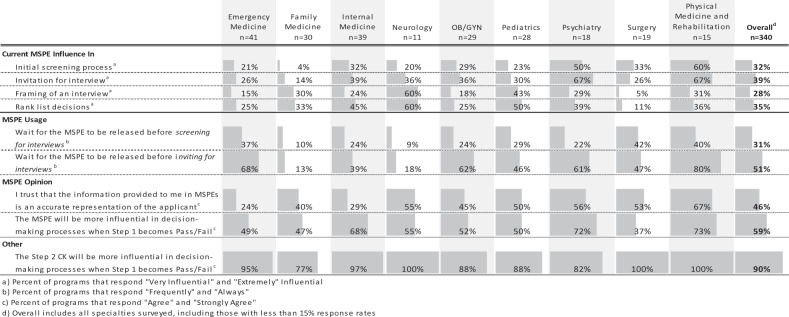

Trust of the MSPE’s ability to accurately represent an applicant varied considerably across specialties (Table 2). Slightly less than half of all end-users agreed or strongly agreed that they trust the information in the MSPE to be an accurate representation of applicants (Table 2). However, even in those specialties with the most distrust, there was considerable value placed on the role it would take on when USMLE Step 1 becomes pass/fail. In addition, almost all end-users also agreed that Step 2 CK will become more influential in the decision-making process when Step 1 scoring becomes pass/fail.

### Areas of importance

The importance of the individual MSPE components to the end user is displayed in (Table 3). The professionalism section rated the highest amongst the readers, who also suggested that negative comments carry the most weight in key decisions of both inviting and ranking applicants (Table 4). The academic progress section has end-users focused on graphic representation of the grade with comments about the grade itself (Table 3).

**Table 3.** Overall and specialty specific attitudes on the importance of each MSPE component. Data presented represents the percent of respondents who chose ‘Very Influential’ and ‘Extremely Influential’ on a 5-point Likert scale

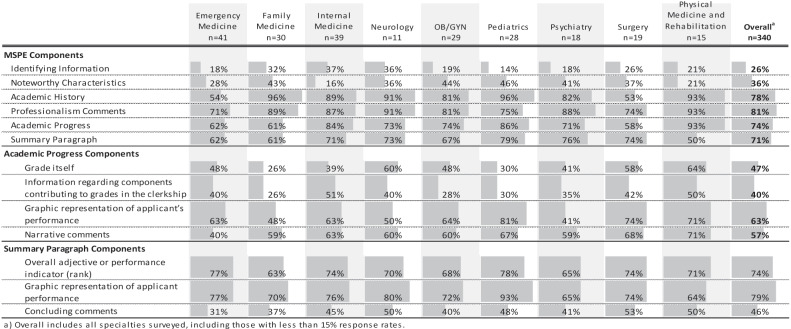

**Table 4.** Overall and specialty specific attitudes on professionalism in the MSPE. Data presented on professionalism components represents the percent of participants that agree that a component should be included in the MSPE. Data on professionalism influence are presented as the percent of respondents who chose the top two highest anchors on a 5-point Likert scale

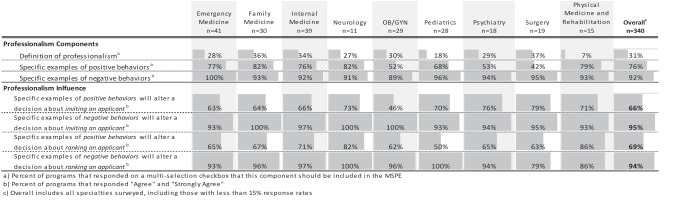

Within the summary paragraph, the majority (79%) of respondents reported that the graphic representation of the applicant’s performance was very or extremely influential. Additionally, most end-users reported that the overall adjective or rank was very or extremely influential. Concluding comments of the MSPE is the least valued of the summary paragraph components (Table 3).

The importance of an applicant’s academic performance in the core clerkships varied by specialty (Table 5). Every specialty ranked performance in their own specialties’ clerkship as most influential (Table 5). However, performance in the IM clerkship was rated as the most influential amongst all specialties.

**Table 5.** Overall and specialty specific attitudes on the importance of an applicant’s academic performance in core clerkships. Data presented represents the percent of respondents who chose ‘Very Influential’ and ‘Extremely Influential’ on a 5-point Likert scale

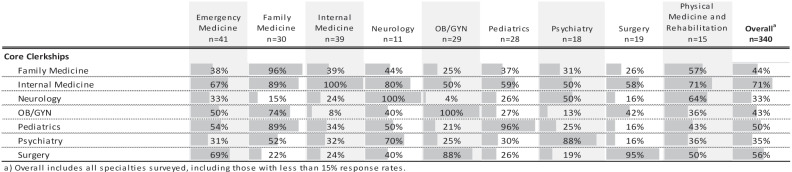

### Suggestions for the future

Recommendations to make the MSPE more useful after USMLE Step 1 score reporting changes included: 1) reporting of comparative performance or class rank (54%); 2) greater transparency including comments addressing areas of improvement (21%); 3) inclusion of more objective measures (e.g., NBME Subject Exam scores) (21%); 4) broader standardization of the template (13%); 5) grades, including reporting of subcomponents (13%). Other suggestions mentioned, but with less frequency (<6%) included a request for earlier release, coinciding with opening of ERAS and the addition of more specific comments about clinical performance, possibly in a framework such as competency, EPA or RIME. The remaining comments were not able to be grouped into a theme due to low frequency.

## Discussion

Despite the AAMC MSPE Task Force guidelines seeking to better standardize the preparation of MSPEs across institutions, this has not necessarily translated into standardized usage. The readers of the MSPE are a diverse group and our findings demonstrate that end-users from different specialties utilize the MSPE for different purposes and at different points along the residency recruitment process, from influencing initial screening of applications, to granting an interview, to preparing for the actual interview, and finally, when creating rank lists.

In terms of the structure of the MSPE, end-users indicated that the noteworthy characteristics were not highly valued with approximately one-third of readers citing it as very or extremely influential. The identification of noteworthy characteristics is sometimes stressful for students, onerous for the MSPE writers, open to implicit bias, and not sufficiently consistent to allow for comparisons of students across schools [15]. The information provided in noteworthy characteristics is available in other components of the application for those who advocate its usefulness as part of a holistic review of applicants [16].

The next section in many MSPEs, the academic history, is a snapshot of the academic program and outlines a timeline of the student’s advancement from matriculation to graduation, making it easy for end-users to identify gaps, adverse events, and remediation; thus it is not surprising that this information was valued to a great extent by respondents, more than double in comparison with noteworthy characteristics. With the overview that the academic history offers, readers are better equipped to search for explanations within the text for any student who diverges from the usual four-year progression.

In many MSPEs, academic history is followed by a statement of the student’s professionalism. In responding to our survey, end-users across specialties identified professionalism as an element in which the MSPE could be most influential, particularly regarding lapses, but also when providing examples of positive behaviors. Our survey confirmed what is likely a fear of many medical school administrators – an acknowledgement that report of lapses could adversely impact a decision about the candidate [17]. This dichotomy likely reflects the inherent tension in the MSPE, where MSPE writers strive to advocate for students and help ‘sell’ their applicants to programs and worry that revealing information about a student’s unprofessional behavior may severely limit that student’s ability to match to a program.

The academic progress section offers readers a combination of grades, grading components, comparative performance, and narrative comments, typically focused on student performance in clerkships. In our survey, the academic progress section was valued more for the narrative comments and graphic representation of a student’s performance than how the grades were derived [16]. This is not surprising since there is a lack of standardization of clerkship grades across institutions and across different clerkships within a single institution, making comparisons extremely difficult, if not impossible [3,4,18–20]. In trying to understand how different specialties view grades in different core clerkships, performance in the IM clerkship held highest value among all specialties with nearly three-quarters of respondents citing performance in IM as being very or extremely influential. Beyond IM, specialties tend to focus on grades in their own specialties or other closely related specialty grades as next most influential, likely based on shared skill sets and/or disease processes and patient problems.

Finally, and in alignment with the literature [21], programs place particular importance on both the overall final adjective and the graphic representation of comparative performance, both thinly veiled surrogates for class rank. Many schools add a summary statement to the adjective and our data indicate that end-users do not find the summary statement to be useful. The summary is not in line with what end-users want, which is objective information, not the school’s interpretation. These concluding comments, which were less valued, have been shown to be highly variable, with evidence of racial and ethnic bias [22,23]. Indeed, the MSPE task force favors omission of the final paragraph, which is more relevant in a letter of *recommendation* than a letter of *evaluation*. Omitting the summary may be a way to entice end-users to read and interpret the entirety of the letter rather than simply the conclusion [22,23].

In looking ahead, respondents overwhelmingly agree that the MSPE will become more influential following the change in USMLE Step 1 score reporting. Coincident with this, our data reveal that many end-users harbor a significant mistrust of the MSPE and have numerous suggestions for inclusion of additional data [24–26]. Until this issue is addressed, it is likely that residency programs will place greater importance on other objective measures (e.g., USMLE Step 2 CK scores, NBME subject exam scores) or on their own internally generated information, such as the EM’s standard letter of evaluation (SLOE) [27,28]. Some of this mistrust may be mitigated with MSPEs that offer more objective information via useful transparent communication about a student’s professionalism, comparative performance indicators such as class rank, and honesty about academic progress, ideally in the body of the MSPE, not in appendices, which require additional searching and scrolling [11,15]. To maximize benefit to the end-user, the medical education community must strive for greater standardization of this document to promote focus on student performance rather than spending unnecessary time deciphering each school’s unique approach. Perhaps thinking of the MSPE as a learner/trainee ‘hand-off’ [24] may make the MSPE more useful to residency programs, about which further research is necessary.

### Limitations

Despite the absolute number of survey respondents of 340, our study is limited by the low overall response rate as well as the variability amongst specialties that responded. Although our numbers per specialty were small, a strength of our study is the wide range of programs which responded, allowing a broad representation of specialties. We opted not to combine specialties into larger groups in order to best represent different approaches to the MSPE use. Our methodology of disseminating the survey to the contact person on the ACGME website was sound but identifying actual readers of the MSPE in each institution remains a challenge.

## Conclusions

Our study is the first to our knowledge to query end-users across a wide breadth of specialties to better understand how they use and value the components of the MSPE. It is also the first survey to look ahead and ask how the MSPE might change once USMLE Step 1 ends three-digit score reporting. End-users across a variety of specialties are a diverse group and utilize this complex letter in different ways and, depending on the specialty, value it differentially in their decision-making processes. Across all specialties, continued mistrust of the MSPE persists. With the impending loss of the USMLE Step 1 score as a discriminating metric, this tension may intensify. A better understanding of end-users’ perceptions of the MSPE offers the UME community an opportunity to transform the MSPE into a highly valued, trusted, and transparent method of communication as desired by the medical education community [24].
